# New ground-based lidar enables volcanic CO_2_ flux measurements

**DOI:** 10.1038/srep13614

**Published:** 2015-09-01

**Authors:** Alessandro Aiuppa, Luca Fiorani, Simone Santoro, Stefano Parracino, Marcello Nuvoli, Giovanni Chiodini, Carmine Minopoli, Giancarlo Tamburello

**Affiliations:** 1 Dipartimento DiSTeM, Università di Palermo, Via Archirafi 36, 90123 Palermo, Italy; 2 Istituto Nazionale di Geofisica e Vulcanologia, Via la Malfa 143, 90146 Palermo, Italy; 3 Diagnostics and Metrology Laboratory, ENEA, Via Enrico Fermi 45, 00044 Frascati, Italy; 4 Guest of the Diagnostics and Metrology Laboratory, ENEA, Via Enrico Fermi 45, 00044 Frascati, Italy; 5 Department of Industrial Engineering, University of Rome “Tor Vergata”, Via del Politecnico 1, 00133 Rome, Italy; 6 Istituto Nazionale di Geofisica e Vulcanologia, Sezione di Bologna, via Donato Creti 12, 40128, Bologna, Italy; 7 Laboratorio di Chimica Ambientale ENEA, Piazzale Enrico Fermi 1, 80055 Portici, Italy

## Abstract

There have been substantial advances in the ability to monitor the activity of hazardous volcanoes in recent decades. However, obtaining early warning of eruptions remains challenging, because the patterns and consequences of volcanic unrests are both complex and nonlinear. Measuring volcanic gases has long been a key aspect of volcano monitoring since these mobile fluids should reach the surface long before the magma. There has been considerable progress in methods for remote and *in-situ* gas sensing, but measuring the flux of volcanic CO_2_—the most reliable gas precursor to an eruption—has remained a challenge. Here we report on the first direct quantitative measurements of the volcanic CO_2_ flux using a newly designed differential absorption lidar (DIAL), which were performed at the restless Campi Flegrei volcano. We show that DIAL makes it possible to remotely obtain volcanic CO_2_ flux time series with a high temporal resolution (tens of minutes) and accuracy (<30%). The ability of this lidar to remotely sense volcanic CO_2_ represents a major step forward in volcano monitoring, and will contribute improved volcanic CO_2_ flux inventories. Our results also demonstrate the unusually strong degassing behavior of Campi Flegrei fumaroles in the current ongoing state of unrest.

Several hundred million people on Earth currently live in the proximity of active or quiescent volcanoes, and therefore could potentially be exposed to the effects of their eruptions. Mitigation of these effects requires careful assessment of volcano behavior and activity state, which can be accomplished via instrument-based volcano monitoring^1^. Recent technological and modeling advances mean that volcanologists can now capture in real time the often-subtle seismic and ground deformation signals that usually precede a volcanic eruption. The advent of broadband seismometers and improved models of and tools for processing seismic signals^2^,^3^,^4^, and the continuous implementation of satellite-based (GPS^5^ and InSaR^6^) geodetic observations have made it possible to record various seismic and deformation signals related to stress changes and magma accumulation/flow that precede an eruption^7^,^8^,^9^,^10^.

The magmatic volatiles^11^ that are continuously released by volcanoes via plumes, fumaroles, and degassing grounds represent the surface manifestation of magma degassing^12^,^13^ at depth, and are therefore a source of potentially invaluable information for predicting the likelihood of a volcano erupting. Spectroscopic techniques working in the ultraviolet (UV) region of the electromagnetic spectrum have recently contributed remotely sensed volcanic SO_2_ flux time series with improved temporal resolution and accuracy^14^,^15^,^16^,^17^, and these data are now facilitating volcanic hazard assessments at several volcano observatories worldwide. However, efforts to remotely detect the flux of volcanic carbon dioxide (CO_2_) have been frustrated by difficulties in distinguishing the volcanic CO_2_ signals from the much-larger background-air signals. Quantification of the volcanic CO_2_ flux has therefore required either time-consuming and expensive airborne CO_2_ plume profiling^18^,^19^,^20^, or hazardous *in-situ* measurement of the volcanic gas CO_2_/SO_2_ ratio via direct sampling^21^,^22^, infrared-red spectrometers^23^,^24^, or gas analyzers^25^,^26^. Soil-gas surveys of hydrothermal volcanoes in a quiescent condition have also quantified diffuse CO_2_ emissions^27^, but much less information has been obtained on fumarolic CO_2_ emissions. Because of these difficulties, the volcanic CO_2_ flux inventory remains sparse and incomplete for most of the active volcanoes on Earth^27^. However, the very few examples of long-term volcanic CO_2_ flux time series have demonstrated prominent peaks in volcanic CO_2_ emission that occur at days to months prior to eruptions^9^,^26^. These have been explained by the low solubility of this gas in silicate melts and its consequent early gas-phase exsolution from ascending (decompressing) magmas. Given the prospects offered by data on the volcanic CO_2_ flux for forecasting eruptions, the ability to remote measure this flux with a high temporal resolution would represent a major advance in modern volcanology.

Here we report on the results of an experiment in which a novel, ad-hoc-designed prototype of a differential absorption lidar (DIAL)^28^ was used to remotely measure the volcanic CO_2_ flux from an active volcano for the first time. Volcano applications of lidars have been limited in the past to measuring the fluxes of volcanic particles^29^, SO_2_^30^, and H_2_O^31^. To the best of our knowledge there has been no previous attempt to measure the volcanic CO_2_ flux using DIAL, although the idea of using DIALs to profile the tropospheric CO_2_ concentration was proposed more than a decade ago^32^. The field experiments we report on here were conducted at Campi Flegrei volcano; this is a resurgent caldera enclosing the city of Pozzuoli and the periphery of Naples, which are two of the most densely populated areas of Italy (Fig. 1). The volcano has been frequently—although discontinuously—active over the past millennia (most recently in 1538 AD^33^,^34^), and is world-renowned for two recent major bradyseismic crises (in 1968–1972 and 1982–1985) that led to a net permanent ground uplift of ~3.3 m and thousands of shallow earthquakes (mostly <4 km deep) felt by the resident population. Although neither crisis evolved into an eruption, each of them illustrated to scientists and stakeholders the potentially severe risk-mitigation issues and the associated required actions^35^. These problems have become even more urgent given that ground uplift resumed in 2012^36^,^37^ accompanied by a visible intensification of degassing activity at the main surface hydrothermal manifestations of Solfatara and Pisciarelli, in the caldera center (Fig. 1). The ongoing uplift/degassing unrest at Campi Flegrei has recently been attributed^37^,^38^ to the periodic injection of CO_2_-rich gas into the volcano’s hydrothermal system, suggesting that new magma is sustaining an intense influx of deep gas. Quantifying the CO_2_ output from the volcanic system is therefore vital to interpreting—and possibly predicting—the future evolution of the volcanic system.

## Results

### Field operations

**Figure 1:**
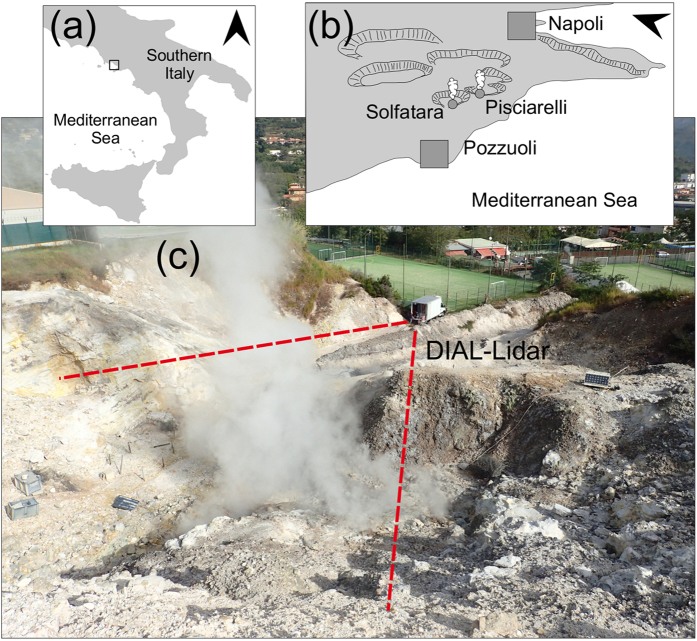
(**a**) A sketch map (created with LibreOffice) of Southern Italy, showing the location of Campi Flegrei caldera (**b**); (**c**) The Pisciarelli fumarolic field, with the DIAL in the background (photo of C. Minopoli).

The DIAL (see Methods; Fig. S1) was mounted on a small laboratory truck and positioned at a fixed location ~100 m from the main degassing fumaroles of Pisciarelli (Fig. 1a). In addition to a subtle increase in discharge temperature (from <95 °C up to 110 °C), the degassing activity at this hydrothermal site has visibly escalated over the past decade^38^, making it the most active fumarolic vent area in Italy^39^ (and possibly in the entire Mediterranean volcanic district). Recurrent episodes of new fumarolic vent opening, associated with the emission of gas and hot mud at high pressures, have also been observed^38^. The fumaroles have also progressively become richer in CO_2_ (CO_2_/H_2_O ratios of >0.3 on a molar basis^37^,^38^), probably indicating an increasingly magmatic nature of the emitted fluids^40^, but also rendering the site ideal for our tests.

**Figure 2:**
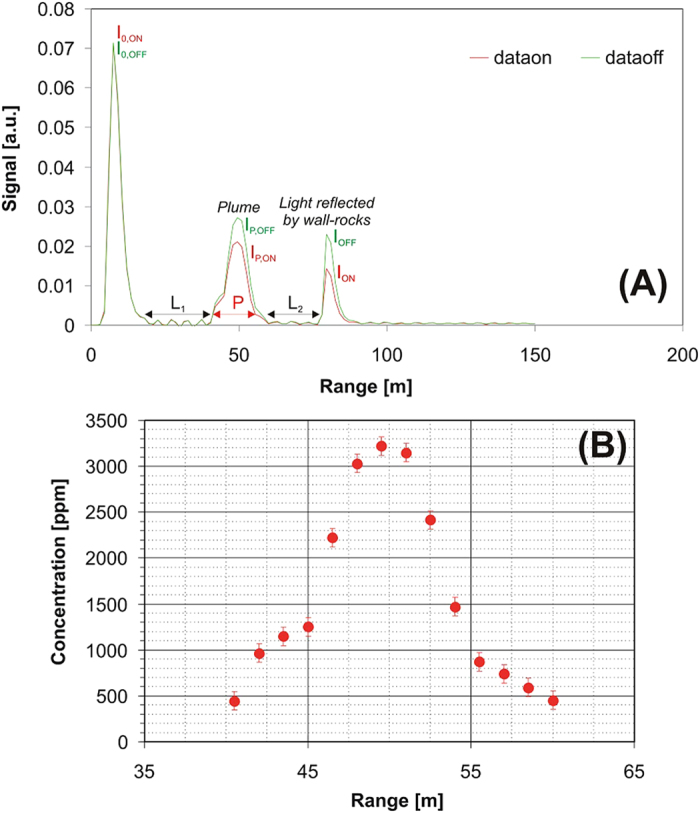
(**A**) An example of a range-resolved lidar signal. The λ_on_ (red) and λ_off_ (green) signals are compared. The three main peaks in the diagram correspond to (from left to right) (i) scattering of some photons of the transmitted laser pulse inside the laboratory truck (this peak gives the exact time of pulse transmission and is proportional to the transmitted energy, thus providing signal normalization), (ii) backscattering of the laser pulse by aerosols in the volcanic plume (mainly water droplets in our case), and (iii) backscattering of the laser pulse off the surface of the rock wall behind the plume (see Figs 1a and S2a). As expected, *I*_0,on_ and *I*_0,off_ are nearly identical, which is due to the wavelengths of the laser signals being very close, and hence are characterized by nearly identical backscattering. In the plume, at about 50 m traveling distance from the laboratory truck, the λ_on_ signal is greatly attenuated due to CO_2_ absorption. This signal attenuation is even greater at the rock-wall surface, after traveling about 80 m. (**B**) Range-resolved calculated in-plume CO_2_ concentrations (see Methods for the technique used to derive concentrations from the lidar signals).

**Figure 3:**
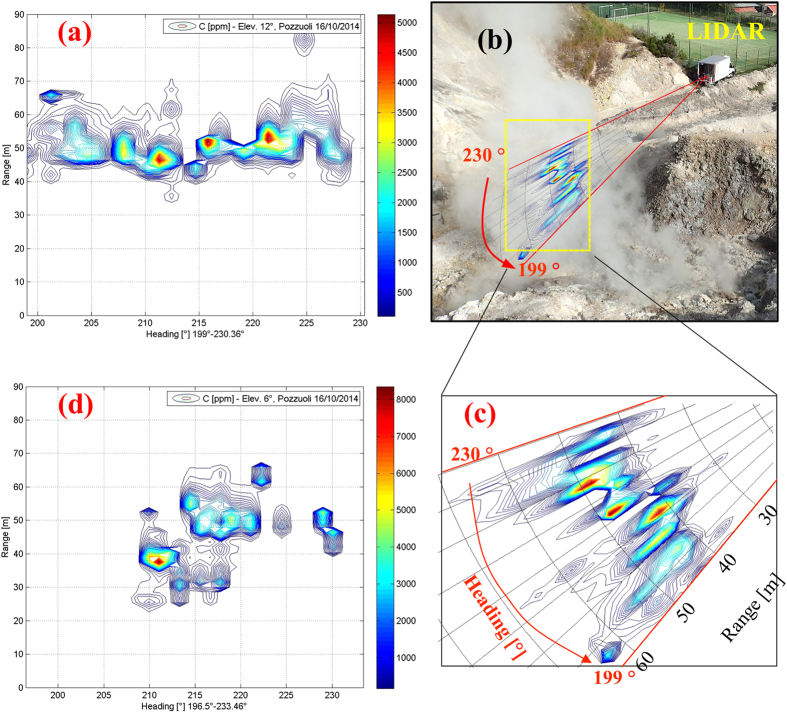
(**a**) An example of a lidar scan through the plume at an elevation of 12° (from October 16, 2014). The contour lines show isopleths of background-corrected CO_2_ mixing ratios in the plume (expressed in ppmv, the legend is the vertical colored bar), shown as a function of heading (horizontal scale) and range (vertical scale). The plume appears as a cluster of CO_2_ concentration peaks at heading angles of 205–225° and ranges of 40 to 60 m from the lidar. At lower (<205°) and higher (>225°) heading angles, and at ranges of <40 and >60 m, CO_2_ mixing ratios corresponding to those for ambient air are obtained. (**b**) Same as (**a**) but in polar coordinates. A photo of the Pisciarelli area (by C. Minopoli) is shown in the background to illustrate the correspondence between the CO_2_ anomaly and the main degassing areas. (**c**) Zoomed image of (**b**), in which the plume is clearly visible at ranges of 40–50 m. (**d**) For comparison, an example lidar scan through the plume at a lower elevation (6°), showing a narrower and less-dispersed plume.

The lidar operated for several consecutive hours during both October 15 and 16, 2014, after the instrument had been set up and prepared for making measurements on October 14. During measurement operations, two large motorized elliptical mirrors were used to horizontally scan the laser beam of the lidar across the vertically rising plume at heading angles from 196° to 230° (Figs 2 and 3). Most of the scans reported herein (Table 1) were performed at an elevation of 12°, at which the plume structure was fully resolved and the lidar signals were strongest. Tests showed that the plume was often either optically too dense or too dispersed at lower and higher elevations, and so only a few additional scans at 0°, 6°, and 15° were successfully completed (Table 1). During each scan, the repetition rate of the laser was set at 10 Hz, but 200 lidar returns (100 at λ_ON_ and 100 at λ_OFF_) were added into a single profile and averaged to increase the signal-to-noise ratio; this meant that the signal sampling frequency was reduced to 0.05 Hz (corresponding to a temporal resolution of 20 s). The spatial resolution was 1.5 m, and a total-plume scan combining 20–30 profiles was retrieved in less than 10 minutes.

### In-plume CO_2_ mixing ratios

The DIAL detected a strong laser absorption signal in the plume (Fig. 2A). Scaling the co-acquired λ_ON_ to the λ_OFF_ signals (composed of 100 additions each), and using the lidar equation (see Methods), the CO_2_ mixing ratios were calculated for each profile (Fig. 2B) and for all profiles of each scan (Fig. 3). Figure 3 illustrates the results of a typical scan through the plume. When the laser beam intercepted the plume, peak CO_2_ mixing ratios of up to 5000 ppmv were measured (Fig. 3a). The plume was typically crossed at heading angles of 205–225° and at ranges (distances) of 40 to 60 m from the lidar. At lower (<205°) and higher (>225°) heading angles, and at ranges of <40 and >60 m, the lidar systematically returned CO_2_ mixing ratios corresponding to those for ambient air, confirming that the entire plume had been crossed by the laser. The polar diagrams, examples of which are given in Fig. 3b,c, show that the peak CO_2_ mixing ratios were observed at heading angles of 210–212° in all of the scans, which directly corresponds to the most vigorously degassing fumarolic vent of Pisciarelli (see Fig. 3c and ref. 39). The same fumarolic vent was even more evident—manifesting as a clear CO_2_ maximum—in scans performed at an elevation angle of 6° (Fig. 3d). The plume appeared to be much narrower at this lower elevation angle, being crossed at heading angles of 210–220°, in agreement with visual observations (Fig. 1). The plume thickness ranged from 5 m close to the main emission vent up to >20 m at the plume margins (Figs 2 and 3).

### Plume transport speed

Quantifying the volcanic CO_2_ flux from our lidar dataset requires knowledge of the speed at which the volcanic gas plume was moving. The vertical plume transport speed was obtained by applying cross-correlation analysis^41^,^42^ to sequences of co-acquired visible images of the moving plume (Fig. S2) that were acquired at a high rate (30 Hz) using a digital optical camera. This procedure (see Methods and Fig. S2) yielded vertical plume transport speeds ranging from 1.4 ± 0.4 (mean ± SD) to 3.0 ± 0.6 m/s (Table 1) at plume heights intercepted by the lidar scans at an elevation of 12° (Fig. S2). Plume speeds of ~10 and 3.0 m/s were calculated for the basal and upper parts of the fumarolic plume, respectively, which were intercepted by some additional lidar scans performed at elevations of 0° and 15° (Table 1). These somewhat higher plume speeds are consistent with vigorous gas jet activity inside the vent (where gas speeds of several tens to hundreds of meters per second have been measured locally in individual spots with centimeter-squared sizes) and at the plume margins (where the convectively rising plume is finally rapidly dispersed by the local wind fields).

### CO_2_ fluxes

**Figure 4:**
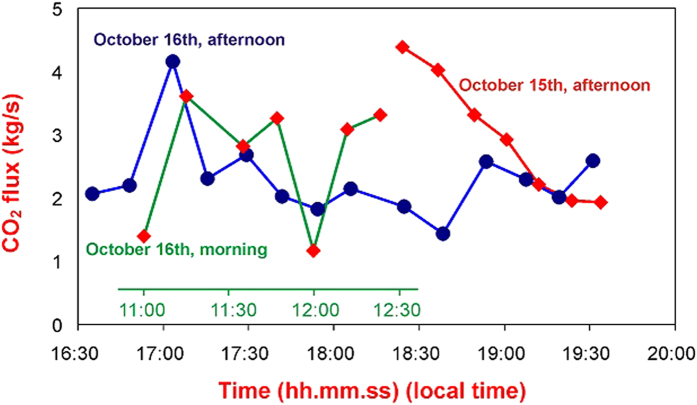
Time series of lidar-derived CO_2_ fluxes from Pisciarelli obtained from October 15 (afternoon) to October 16 (afternoon). The timescale for the morning scans on October 16 is shown in green. Each point refers to a particular scan through the plume (time is the onset time of the scan, with each scan lasting 10–20 minutes).

The CO_2_ flux from the fumarolic system was obtained by integrating the background-corrected CO_2_ mixing ratios over the entire plume cross-sectional area covered by each scan, and multiplying this integrated amount in the column by the plume transport speed (see Methods). This procedure made it possible to produce CO_2_ flux time series with a high temporal resolution (10–20 minutes), which revealed large intraday variations (Table 1, Fig. 4) that would have been difficult to detect using conventional techniques. The time series in Fig. 4 reveal that the CO_2_ flux at the Pisciarelli hydrothermal site can exhibit dramatic variations over timescales of tens of minutes, such as from a peak emission of 4.4 kg/s (at 18:23 hours local time on October 15) down to 1.9 kg/s less than 1 hour later. Apart from these rapid fluctuations, our results support an overall stability of time-averaged volcanic CO_2_ emissivity, at least over timescales of days. The time-averaged CO_2_ outputs for the two measuring days (October 15 and 16, 2014) are highly consistent, at 2.63 ± 0.98 and 2.52 ± 0.84 kg/s (Table 1), and correspond to total daily outputs of 256 ± 89 and 218 ± 71 tons of CO_2_, respectively.

## Discussion

Previous attempts to directly measure the volcanic CO_2_ flux using remote sensing techniques have been frustrated by the much larger amount of atmospheric CO_2_ present along the optical path between the source of radiation (either the sun, the magma, or molten lava fragments), the target (the volcanic plume), and the measuring spectrometer. The DIAL technique, which is based on measuring the intensity of the irradiation from a laser beam backscattered by atmospheric scatterers (particles, aerosols, and water droplets)^43^, does not require the use of external light sources, and therefore offers range-resolved (Figs 2 and 3) high-precision CO_2_ sensing over long atmospheric optical paths^44^,^45^,^46^. Despite this advantage, DIALs have not been given much consideration for volcanic CO_2_ detection until recently, partly because of the relatively large cost, weight, and size of the instrumentation.

We have here described a 2-μm DIAL whose high spatial resolution makes it possible to characterize the geometry, 2D structure, and CO_2_ column density of a volcanic gas plume (Fig. 3). Figure 3 shows two 2D scans of the plume obtained at different elevations (6° and 12°). The large contrast between the lidar signals returned from the plume and the ambient air (Figs 2 and 3) makes it possible to distinguish the volcanic CO_2_ contribution from the background CO_2_ signal, and therefore allows a robust quantification of the volcanic CO_2_ flux—after integrating the volcanic CO_2_ concentrations over the entire cross section of the plume and multiplying this by the plume transport speed.

**Figure 5:**
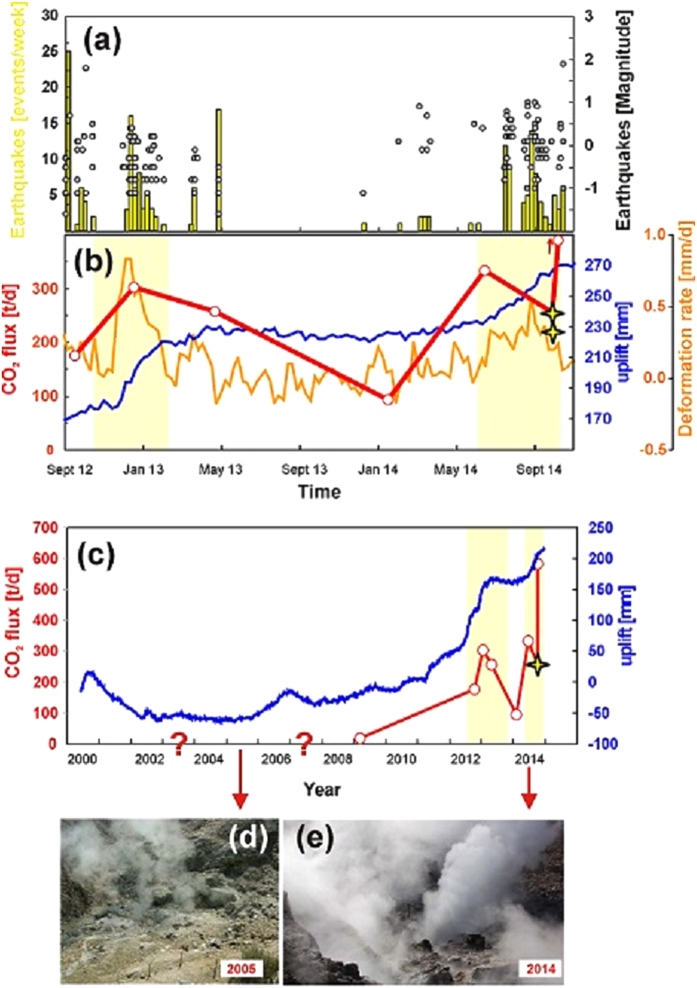
The Campi Flegrei unrest, as illustrated by time series of (a) seismicity (number of events/week and event magnitude between September 2012 and December 2014; data from Osservatorio Vesuviano, INGV) and (b) ground uplift (blue line; uplift during 2012–2014 in millimeters, with the ground reference level obtained in January 2005) and deformation rate (orange line; monthly time average, expressed in millimeters per day) (data from ref. 36 and Osservatorio Vesuviano, INGV). The CO_2_ flux time series (red line) is also shown in (**b**) (stars: DIAL dataset, this work; open circles, derived from in-plume Multi-GAS profiling^32^; data from ref. 32 and this study). (**c**) Long-term (2000–2014) temporal evolution of ground uplift (blue, data from ref. 36 and Osservatorio Vesuviano, INGV). The CO_2_ flux time series also includes the results from a survey carried out in March 2009 (data from Osservatorio Vesuviano, INGV), which was prior to the recent intensification of degassing activity at Pisciarelli. The visible escalation of degassing activity at Pisciarelli is evident from comparison of images obtained in 2005 (d, photo of G. Chiodini) and 2014 (e, photo of G. Tamburello).

The lidar-based CO_2_ fluxes we have measured at Pisciarelli (Table 1) are in good overall agreement with previous CO_2_ flux estimations (Fig. 5) based on *in-situ* measurements of CO_2_ concentrations in the plume made using a portable gas analyzer^39^. However, the lidar offers substantial advantages over these more traditional techniques. The remote-sensing nature of using the lidar makes it intrinsically safer for operators when access to direct sampling of gas manifestations becomes hazardous, or even impossible, due to intensification of degassing activity close to the time of an eruption. As such, our novel lidar instrument could also assist systematic volcano CO_2_ flux observations even during volcanic eruptions; such measurements are virtually missing from the geological literature, and they would be very useful. Our lidar also provides a substantial improvement in measurement frequency, with the temporal resolution of our CO_2_ flux observations (10–20 minutes) being much higher than that of both conventional^39^ and recently developed^47^ techniques, whose temporal resolutions are typically on the order of several hours or even days. We therefore conclude that the advent of DIALs could lead to major advances in the understanding of volcanic degassing processes, and of their implications for eruption forecasting.

Our measurements also provide new information for bettering understanding the causes and potential consequences of the ongoing degassing and deformation unrest at Campi Flegrei (Fig. 5). The lidar-based CO_2_ flux dataset provides additional, independent evidence for the unusually intense CO_2_ degassing behavior at Campi Flegrei in general, and the Pisciarelli hydrothermal area in particular. Figure 5 shows that Pisciarelli fumaroles currently sustain a persistent daily CO_2_ output of 100–350 tons, which is disproportionately high for a geothermal area of only ~1000 m^2^, but is more similar to the typical intereruptive CO_2_ emissions from a recurrently active arc volcano, such as Merapi in Indonesia (240 tons/day)^48^. For comparison, the CO_2_ flux in the Pisciarelli area was evaluated at only ~18 tons/day in March 2009 (Osservatorio Vesuviano, internal reports), prior to the recent period of intensification of ground uplift (Fig. 5c). The origin of the CO_2_ emitted by Campi Flegrei remains debated due to its rather equivocal carbon-isotope signature, which is intermediate between magmatic and crustal isotopic domains^40^. However, there is convincing evidence from the chemistry of fumarolic gases^37^,^38^ that the visible escalation in degassing activity, and possibly the ground uplift itself, are caused by periodic injections of magmatic gas into the Campi Flegrei hydrothermal system^37^, with a recurrence time that has been reducing since 2005. A progressive heating of the hydrothermal system, possibly initiated by the increasing H_2_O content of the magmatic gas supply, has also been proposed recently^38^. If this hypothesis is correct, it has a corollary that the ongoing unrest, including acceleration of the CO_2_ degassing regime, has a magmatic trigger^39^,^49^. A cause–effect link between deformation and magmatic degassing has been proposed^37^,^38^ based on the temporal coincidence between the acceleration of ground uplift and peaks in magmatic gas proxies (e.g., CO_2_/CH_4_ ratios^50^) in fumaroles. Our new results presented in Fig. 5 extend previous conclusions by documenting that an extensive parameter, such as the fumarolic CO_2_ flux, also co-varies with deformation and seismicity. Although the gas flux time series is far more discontinuous than the regularly monitored geophysical signals, the available data still support the presence of correlations between peaks in the CO_2_ flux (>200 tons/day) and episodes of ground uplift (with deformation rates >+0.25 mm/day) (Fig. 5b) and seismicity (Fig. 5a). The lowest CO_2_ flux (<100 tons/day) in our dataset was typically observed during a seismically silent period (in early 2014), when there was also a pause in the deformation. The overall long-term escalation of both degassing and deformation (Fig. 5c) raises concern about the future evolution of the Campi Flegrei unrest, and indicates that monitoring efforts need to be intensified.

The DIAL, with its ability to remotely sense volcanic CO_2_, is a new tool in the armoury of volcanologists. However, a considerable amount of further development is needed before this technology can become an operational tool for use in routine volcanic gas observations. The prototype presented here (Fig. S1) has limited portability (weighing 1100 kg and having dimensions of 3.4 × 2.1 × 2.0 m^3^), relatively high power requirements (6.5 kW), and requires further modifications before fully unattended and continuous observations will be possible. Future developments include miniaturization and automation, which will require a new generation of more-compact narrowband pulsed laser sources. A more-compact lidar that has a lower power requirement and is based on an optical parametric oscillator source is currently being testing in our laboratories. In order to make lidars of wider utility for volcano applications, the next stages of development will also require adaptation to longer measurement distances than those described here (~100 m). Our preliminary tests show that the atmospheric CO_2_ profile over paths up to 2 km long can be measured with the present system, but it remains unclear whether it will be possible to distinguish a volcanic signal from the atmospheric CO_2_ over such long measurement distances. An additional limitation is that our lidar is not measuring a flux itself, but rather the CO_2_ distribution in a cross section of the plume (Figs 2 and 3). This implies that an independent method for determining the plume speed is still needed before the CO_2_ flux can be calculated (videography was used in the present study). Techniques to measure plume speed can be implemented in lidars, including a correlation technique^31^ or by using Doppler lidars^51^. Although the possibility was not been fully explored in the present study, lidars can also be used to characterize the 3D structure of a volcanic plume, by combining several 2D scans performed at different elevations. These examples illustrate the considerable prospects for lidar systems in assisting volcanic CO_2_ flux observations in the future.

## Methods

### The DIAL technique

The DIAL^43^ is based on transmitting laser pulses at two distinct wavelengths: one corresponding to the absorption line of the element of interest, λ_on_, and another at a nearby wavelength, λ_off_. Assuming equal scattering by molecules and particles at the two wavelengths, the difference in the backscattered radiation at λ_on_ and λ_off_ is only due to absorption by molecules of the target gas. CO_2_ detection by DIAL has been suggested in the 2.0 and 1.6 μm bands^32^, and Tm:Ho:YLF lasers^45^ and fiber lasers^46^ have recently been employed for DIAL-based sensing of atmospheric CO_2_. Dye-laser-based systems^52^ are tunable over a wide range, from UV to near-infrared, which offers the advantage of targeting several distinct CO_2_ absorption lines. The application of lidars to atmospheric CO_2_ detection has also been described recently^53^.

### The “BRIDGE” DIAL

We used a dye-laser-based DIAL^28^ developed as part of the “BRIDGE” project funded by the European Research Council. This laser system was selected due to the circular profile, small size, and low divergence of its laser beam. The transmitter (Fig. S1 and Table S1) is composed of (i) an injection-seeded Nd:YAG laser, (ii) a double-grating dye laser, (iii) a difference-frequency mixing (DFM) device, and (iv) an optical parametric amplifier (OPA). The circular cross section of the amplifier cells inside the dye laser means that the beam intensity profile in the far field is close to a Gaussian distribution. The dye laser resonator has a longitudinal mode spacing of 0.018 cm^−1^ and a line width of 0.025 cm^−1^ at 696 nm, which is used to generate laser radiation at 2012 nm. This latter line was selected given its high CO_2_ absorption coefficient but minimal cross-sensitivity to H_2_O^54^. The line width is larger than the spacing, and so two longitudinal modes can be emitted. However, since the energy can be distributed in unknown and unequal parts between the two modes, a dynamic mode option was implemented: a piezoelectric element is used to change the resonator length of the dye laser at each shot, thereby modifying the longitudinal mode structure of successive pulses and allowing for the spectral shape to be statistically distributed (in our case 50% of the energy is emitted in each mode). The laser system can be switched from λ_ON_ to λ_OFF_ at a repetition rate of 10 Hz by using piezoelectric-element-based wavelength control (Fig. S1). This was made possible by mounting the rotatable Littrow grating of the dye laser (which is usually controlled by a relatively slow stepper motor) on a tilting piezoelectric element. However, since the nonlinear crystals of the DFM device and the OPA cannot move at a 10-Hz repetition rate, two additional nonlinear crystals were mounted (at a slightly different angle): one after the standard mixing crystal and the second after the standard amplifier crystal. The receiver of the lidar is based on a Newtonian telescope. A thermoelectrically cooled InGaAs PIN photodiode, directly connected to the analog-to-digital converter (ADC), was used as a detector. Two large elliptical mirrors (major axis: 450 mm) allow the lidar to scan the entire hemisphere above the horizon. The wavelength accuracy, repeatability, and stability of the complete system were tested with a cell filled with CO_2_^55^.

### Retrieval of CO_2_ concentrations

Each path of the laser beam can be divided into two portions: inside and outside the plume. The latter portion includes two segments, before (length: *L*_1_) and after (length: *L*_2_) passing through the plume (see Fig. 2a). Assuming that the CO_2_ concentration inside the plume (*P* in Fig. 2a) is proportional to the lidar signal (which is, in turn, proportional to the aerosol load, and is dominated by water droplets in our case), then the optical depth (OD) of the path of the laser beam can be written as
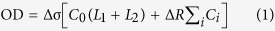
where Δσ is the CO_2_ differential absorption cross section, *C*_0_ is the CO_2_ concentration in the natural atmospheric background outside the plume (=400 ppmv), Δ*R* is the ADC range resolution, and *C*_*i*_ is the CO_2_ concentration corresponding to the *i*-th ADC channel (inside the plume). This scales to the corresponding lidar off signal *S*_*i*_ in the *i*-th ADC channel according to
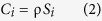
where ρ is a proportionality constant. The value of ρ, and hence also of *C*_*i*_, can be retrieved because Δσ, *C*_0_, and Δ*R* are known, and OD, *L*_1_, *L*_2_ and *S*_*i*_ are measured from the lidar signal. More precisely, OD is obtained from the peaks of the ON and OFF lidar signals caused by reflection of the laser beam in the laboratory truck (*I*_0_,_ON_ and *I*_0_,_OFF_) and off the surface of the rock wall behind the plume (*I*_ON_ and *I*_OFF_) (see Fig. 2a):
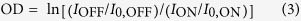
and *S*_*i*_ is the lidar signal in the *i*-th ADC channel inside the plume.

The measurement error was evaluated by operating the lidar in air free from CO_2_ sources, both at the Frascati ENEA research center and in Pisciarelli (with the laser beam aimed far from the plume). These tests demonstrate a relative error in the retrieved CO_2_ concentrations of 1–10%, which corresponds to a maximum error of 100 ppmv over the range of volcanic CO_2_ concentrations of 10,000 to 1000 ppmv encountered in this study.

### Plume transport speed

We processed sequences of visible images of the fumarolic field (Fig. S2a) acquired at 30 Hz using a digital optical camera to calculate the transport speed of the condensed volcanic gas plume. The procedure is based on an analysis of the brightness of the digital images, where each pixel has a brightness value ranging from 0 to 1. Pixels corresponding to the volcanic plume are much brighter than pixels of the atmospheric background around the plume itself (Fig. S2b). This indicates that a large fraction of the “in-plume” luminance received by the optical sensor of the camera is radiation scattered by the condensed gas plume, which is in turn proportional to the time- and space-dependent concentrations of condensed volcanic water (e.g., water droplets)^56^. The brightness of the background pixels was reasonably constant over timescales of minutes to hours. Integration over two parallel, closely spaced brightness profiles through the rising plume (Fig. S2c) results in two “integrated brightness” time series, with a temporal shift Δ*t* (Fig. S2d) corresponding to the time that the gas cloud takes to travel from the first to the second section (a similar procedure has been applied previously to the UV region of the electromagnetic spectrum^41^,^42^). Time series of the temporal shift Δ*t* were calculated via cross-correlation analysis with a moving window, and corresponded to shift values at which the maximum correlation coefficients (*R*^2^) between the two time series were obtained. The obtained Δ*t* values were finally converted into time series of the plume transport speed (Fig. S2e) multiplied by the spacing (Δ*x*) between the two profiles. The smoothness and accuracy of the derived velocity time series depend on the window length and acquisition rate of the images^42^. At 30 Hz, we found that a 30-s window is adequate for our purposes. Since the digital camera and the lidar were not perfectly synchronized during the operations, we report in Table 1 the mean plume speeds calculated for 30-minute-long temporal intervals, overlapping with the lidar scans. Note that the plume speeds were calculated for different levels of the plume (obtained by moving the positions of the *j* and *k* sections) for consistency with the different elevation angles (0–15°) of the lidar scans.

### Calculation of the CO_2_ flux

The CO_2_ flux (ΦCO_2_, in kilograms per second) was obtained by multiplying the vertical plume transport speed (*v*_P_) by the total-plume CO_2_ molecular density (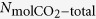, expressed in molecules per meter), according to
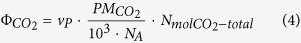
where 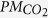 and *N*_A_ are the molecular weight and Avogadro’s constant, respectively, and 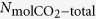 is obtained after integrating the background-corrected CO_2_ concentrations (*C*_*i*-corr_) over the entire plume cross section, according to
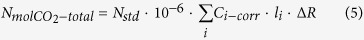
where *N*_std_ is the Loschmidt number (2.46 × 10^25^ molecules/m^3^; or the molecular atmospheric density per unit volume at standard temperature and pressure), Δ*R* is the spatial resolution of the lidar (1.5 m), and *l*_*i*_ is the *i*-th arc of circumference (see Fig. S3), or
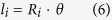
where 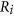 is the *i*-th distance vector (in meters) and 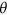 is the angular resolution of the system (=1.12°) expressed in radians (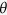 = 1.12° · π/180 = 0.1955 rad) (see Fig. S3).

The relative error of Σ_*i*_C_*i*-corr_ is a function of the relative error of C_*i*-corr_, and is estimated to be about 5%. This, combined with an overall uncertainty in the derived plume speeds of 10–28%, leads to a cumulative error in the calculated CO_2_ fluxes of ~15–33%.

## Additional Information

**How to cite this article**: Aiuppa, A. *et al.* New ground-based lidar enables volcanic CO_2_ flux measurements. *Sci. Rep.* **5**, 13614; doi: 10.1038/srep13614 (2015).

## Supplementary Material

Supplementary Information

## Figures and Tables

**Table 1:** Results of successful DIAL scans.

October 15					
Time [HH:MM:SS]	Plume speed [m/s]	Elevation [°]	Scan no.	Flux [kg/s]	Flux [tons/day]
18:23:07	1.4 ± 0.4	12	8	4.4 ± 1.5	379 ± 127
18:35:00	1.4 ± 0.4	12	9	4.0 ± 1.3	347 ± 116
18:47:18	1.4 ± 0.4	12	10	3.3 ± 1.1	286 ± 96
18:58:26	1.4 ± 0.4	12	11	2.9 ± 1.0	252 ± 84
19:09:18	1.4 ± 0.4	12	12	2.2 ± 0.7	191 ± 64
19:20:20	1.4 ± 0.4	12	13	2.0 ± 0.7	170 ± 57
19:30:08	1.4 ± 0.4	12	14	1.9 ± 0.6	167 ± 56
**Mean (**±1 SD)				**2.6** ± **1.0**	**256** ± **85**
**October 16**					
10:56:02	3.0 ± 0.5	15	3	1.5 ± 0.3	131 ± 27
11:10:09	3.0 ± 0.5	15	4	3.9 ± 0.8	338 ± 71
11:29:17	1.5 ± 0.3	12	5	3.2 ± 0.8	274 ± 66
11:40:44	1.5 ± 0.3	12	6	3.7 ± 0.9	316 ± 76
11:53:03	1.5 ± 0.3	12	7	1.3 ± 0.3	113 ± 27
12:04:40	1.5 ± 0.3	12	8	3.5 ± 0.8	299 ± 72
12:15:47	1.5 ± 0.3	12	9	3.7 ± 0.9	321 ± 77
16:38:30	10 ± 1	0	13	2.1 ± 0.3	178 ± 27
16:51:09	10 ± 1	0	14	2.2 ± 0.3	190 ± 28
17:05:30	3.0 ± 0.6	6	15	4.2 ± 1.0	359 ± 90
17:17:22	3.0 ± 0.6	6	16	2.3 ± 0.6	199 ± 50
17:30:37	3.0 ± 0.6	12	17	2.7 ± 0.7	231 ± 58
17:42:23	3.0 ± 0.6	12	18	2.0 ± 0.5	174 ± 43
17:54:24	3.0 ± 0.6	12	19	1.8 ± 0.5	158 ± 39
18:05:33	3.0 ± 0.6	12	20	2.1 ± 0.5	185 ± 46
18:23:47	3.0 ± 0.6	12	21	1.9 ± 0.4	161 ± 40
18:36:58	3.0 ± 0.6	12	22	1.4 ± 0.6	124 ± 31
18:51:23	3.0 ± 0.6	12	23	2.6 ± 0.6	223 ± 55
19:04:59	3.0 ± 0.6	12	24	2.3 ± 0.5	198 ± 49
19:16:08	3.0 ± 0.6	12	25	2.0 ± 0.6	174 ± 43
19:27:33	3.0 ± 0.6	12	26	2.6 ± 0.5	224 ± 56
**Mean (**±1 SD)				**2.5** ± **0.8**	**218** ± **71**

A scan was considered successful when observations were performed across the entire plume cross section (e.g., when background values were obtained in lidar profiles obtained at heading angles of <200° and >225°). This condition was fully met during phases of a buoyant, vertically ascending plume when the effect of the external (local) wind pattern was either minor or negligible. Each CO_2_ flux value was obtained by integrating the background-corrected CO_2_ mixing ratios over the entire plume cross-sectional area covered by each scan, and multiplying this integrated amount in the column by the plume transport speed (see Methods). The plume speeds and fluxes are presented as mean ± SD values.

## References

[b1] Sparks R. S. J., Biggs J. & Neuberg J. W. Monitoring Volcanoes. Science 335, 1310; 10.1126/science.1219485 (2012). 22422969

[b2] Chouet B. Long-period volcano seismicity: its source and use in eruption forecasting. Nature 380, 309 – 316, 10.1038/380309a0 (1996).

[b3] Neuberg J. W. Earthquakes, Volcanogenic in *Encyclopaedia of Solid Earth* Geophysics, Vol. 1 (ed. Gupta H. K. ), 261–269 (Springer, Berlin/Heidelberg, 2011).

[b4] Chouet B. A. & Matoza R. S. A multi-decadal view of seismic methods for detecting precursors of magma movement and eruption. J. Volcanol. Geotherm. Res. 252, 108–175 (2013).

[b5] Dzurisin D. A comprehensive approach to monitoring volcano deformation as a window on the eruption cycle. Rev. Geophys. 41, 1001 (2003).

[b6] Biggs J. *et al.* Global link between deformation and volcanic eruption quantified by satellite imagery. Nature Communications 5, 10.1038/ncomms4471 (2014). PMC4409635 24699342

[b7] Iverson R. M. *et al.* Dynamics of seismogenic volcanic extrusion at Mount St Helens in 2004–05. Nature 444, 439–443 (2006). 17122849 10.1038/nature05322

[b8] Sigmundsson F. *et al.* Intrusion triggering of the 2010 Eyjafjallajökull explosive eruption. Nature 468, 426–430, 10.1038/nature09558 (2010). 21085177

[b9] Poland M. P., Miklius A., Sutton A. J. & Thornber C. R. A mantle-driven surge in magma supply to Kīlauea Volcano during 2003–2007. Nature Geoscience 5, 295–300, 10.1038/ngeo1426 (2012).

[b10] Sigmundsson F. *et al.* Segmented lateral dyke growth in a rifting event at Bárðarbunga volcanic system, Iceland. Nature 517, 191–195, 10.1038/nature14111 (2014). 25517098

[b11] Aiuppa A. Volcanic gas monitoring. in Volcanism and Global Environmental Change (eds Schmidt A., Fristad K. E. & Elkins-Tanton L. T. ) (Cambridge University Press, 2015).

[b12] Oppenheimer C. Fischer T. & Scaillet B. Volcanic Degassing: Process and Impact in *Treatise on Geochemistry* (Second Edition), (ed. Holland H. D. & Turekian K. K. ), 111–179, 10.1016/B978-0-08-095975-7.00304-1 (Elsevier, Oxford, 2014).

[b13] Edmonds M. New geochemical insights into volcanic degassing. Phil. Trans. R. Soc. A 366, 4559 (2008). 18826919 10.1098/rsta.2008.0185

[b14] Edmonds M. *et al.* Automated, high time-resolution measurements of SO_2_ flux at Soufrière Hills Volcano, Montserrat. Bull. Volcanol. 65, 578–586 (2003).

[b15] Galle B. *et al.* Network for Observation of Volcanic and Atmospheric Change (NOVAC) – A global network for volcanic gas monitoring: Network layout and instrument description. J Geophys. Res. 115, D05304 (2010).

[b16] Oppenheimer C., Scaillet B. & Martin R. S. Sulfur degassing from volcanoes: source conditions, surveillance, plume chemistry and impacts. Reviews in Mineralogy and Geochemistry 73, 363–421, 10.2138/rmg.2011.73.13 (2011).

[b17] Mori T. & Burton M. The SO_2_ camera: A simple, fast and cheap method for ground-based imaging of SO_2_ in volcanic plumes. Geophysical Research Letters 33, L24804, 10.1029/2006GL027916 (2006).

[b18] Allard P. *et al.* Eruptive and diffuse emissions of CO_2_ from Mount Etna. Nature 351, 387–391 (1991).

[b19] Gerlach T. M. *et al.* Application of the LI-COR CO_2_ analyser to volcanic plumes: a case study, volcan Popocatepetl, Mexico, June 7 and 10, 1995. J. Geophys. Res. 102, B4, 8005–8019 (1997).

[b20] Werner C. *et al.* Deep magmatic degassing versus scrubbing: elevated CO_2_ emissions and C/S in the lead-up to the 2009 eruption of Redoubt volcano, Alaska. Geochem. Geophys. Geosyst. 13, Q03015 (2012).

[b21] Hilton D. R., Fischer T. P. & Marty B. Noble gases and volatile recycling at subduction zones, Rev. Mineral. Geochem. 47, 319–370 (2002).

[b22] Fischer T. P. Fluxes of volatiles (H_2_O, CO_2_, N_2_, Cl, F) from arc volcanoes. Geochem. J. 42, 21–38 (2008).

[b23] Burton M. R., Oppenheimer C., Horrocks L. A. & Francis P. W. Remote sensing of CO_2_ and H_2_O emission rates from Masaya Volcano, Nicaragua. Geology 28, 915–918 (2000).

[b24] Oppenheimer C. & Kyle P. R. Probing the magma plumbing of Erebus volcano, Antarctica, by open-path FTIR spectroscopy of gas emissions. J. Volcanol. Geotherm. Res. 177(3), 743–754, 10.1016/j.jvolgeores.2007.08.022 (2008).

[b25] Aiuppa A., Federico C., Giudice G. *et al.* (2006). Rates of carbon dioxide plume degassing from Mount Etna volcano. J. Geophys. Res. 111, B09207, 10.1029/2006JB004307

[b26] Aiuppa *et al.* Unusually large magmatic CO_2_ gas emissions prior to a basaltic paroxysm. Geophys. Res. Lett. 37 (17), art. no. L17303 (2010).

[b27] Burton M. R., Sawyer G. M. & Granieri D. Deep carbon emissions from volcanoes. Reviews in Mineralogy and Geochemistry 75(1), 323–354 (2013).

[b28] Fiorani L. *et al.* Volcanic CO_2_ detection with a DFM/OPA-based lidar. Opt. Lett. 40, 1034–1036 (2015). 25768175 10.1364/OL.40.001034

[b29] Fiorani L., Colao F. & Palucci A. Measurement of Mount Etna plume by CO_2_-laser-based lidar. Opt. Lett. 34, 800–802 (2009). 19282937 10.1364/ol.34.000800

[b30] Weibring P. *et al.* Monitoring of volcanic sulphur dioxide emissions using differential absorption lidar (DIAL), differential optical absorption spectroscopy (DOAS), and correlation spectroscopy (COSPEC). Appl. Phys. B 67, 419–426 (1998).

[b31] Fiorani L. *et al.* First-time lidar measurement of water vapor flux in a volcanic plume. Opt. Comm. 284, 1295–1298 (2011).

[b32] Menzies R. T. & Tratt D. M. Differential laser absorption spectrometry for global profiling of tropospheric carbon dioxide: selection of optimum sounding frequencies for high-precision measurements. Appl. Opt. 42, 6569–6577 (2003). 14658457 10.1364/ao.42.006569

[b33] Orsi G., Di Vito M. A. & Isaia R. Volcanic hazard assessment at the restless Campi Flegrei caldera. Bull. Volcanol. 66, 514–530 (2004).

[b34] Orsi G., Di Vito M. A., Selva J. & Marzocchi W. Long-term forecast of eruptive style and size at Campi Flegrei caldera (Italy). Earth Planet. Sci. Lett. 287, 265–276 (2009).

[b35] Barberi F., Corrado G., Innocenti F. & Luongo G. Phlegraean Fields 1982-1984: brief chronicle of a volcano emergency in a densely populated area. Bull. Volc. 47-2, 175–185 (1984).

[b36] De Martino P., Tammaro U. & Obrizzo F. GPS time series at Campi Flegrei caldera (2000-2013). Annals of geophysics 57, 2, S0213; 10.4401/ag-6431 (2014).

[b37] Chiodini G., Caliro S., De Martino P., Avino R. & Ghepardi F. Early signals of new volcanic unrest at Campi Flegrei caldera? Insights from geochemical data and physical simulations. Geology 40, 943–946 (2012).

[b38] Chiodini G. *et al.* Evidence of thermal-driven processes triggering the 2005–2014 unrest at Campi Flegrei caldera. Earth and Planetary Science Letters 414, 58–67 (2015).

[b39] Aiuppa A. *et al.* First observations of the fumarolic gas output from a restless caldera: Implications for the current period of unrest (2005–2013) at Campi Flegrei. Geochem. Geophys. Geosys. 14, 4153–4169 (2013).

[b40] Caliro S., Chiodini G. *et al.* The origin of the fumaroles of La Solfatara (Campi Flegrei, south Italy). Geochim. Cosmochim. Acta 71(12), 3040–3055, 10.1016/j.gca.2007.04.007 (2007).

[b41] Tamburello G. *et al.* Passive vs. active degassing modes at an open-vent volcano (Stromboli, Italy). Earth Planet. Sci. Lett. 359–360, 106–116 (2012).

[b42] Boichu M., Oppenheimer C., Tsanev V., Kyle P. R High temporal resolution SO_2_ flux measurements at Erebus volcano, Antarctica. J. Volcanol. Geotherm. Res. 190, 325–336 (2010).

[b43] Fiorani L. Lidar application to lithosphere, hydrosphere and atmosphere in Progress in Laser and Electro-Optics Research (ed. Koslovskiy V. V. ), 21–75 (Nova, New York, 2010).

[b44] Gibert F., Flamant P. H., Cuesta J. & Bruneau D. Vertical 2-μm heterodyne differential absorption lidar measurements of mean CO_2_ mixing ratio in the troposphere. J. Atm. Ocean. Tech. 25, 1478–1497 (2008).

[b45] Koch G. J. *et al.* Side-line tunable laser transmitter for differential absorption lidar measurements of CO_2_: design and application to atmospheric measurements. Appl. Opt. 47, 944–956 (2008). 18311266 10.1364/ao.47.000944

[b46] Kameyama S. *et al.* Development of 1.6 μm continuous-wave modulation hard-target differential absorption lidar system for CO_2_ sensing. Opt. Lett. 34, 1513–1515 (2009). 19448805 10.1364/ol.34.001513

[b47] Pedone M. *et al.* Volcanic CO_2_ flux measurement at Campi Flegrei by Tunable Diode Laser absorption Spectroscopy. Bull. Volc. 76, 10.1007/s00445-014-0812-z (2014).

[b48] Toutain J.-P. *et al.* Structure and CO_2_ budget of Merapi volcano during inter-eruptive periods. Bull Volcanol 71(7), 815–826, 10.1007/s00445-009-0266-x (2009).

[b49] Caliro S., Chiodini G., Paonita A. Geochemical evidences of magma dynamics at Campi Flegrei (Italy). Geochimica et Cosmochimica Acta 132, 1–15 (2014).

[b50] Chiodini G. CO_2_/CH_4_ ratio in fumaroles a powerful tool to detect magma degassing episodes at quiescent volcanoes. Geophysical Research Letters 36 (2), art. no. L02302 (2009).

[b51] Menzies R. T. Doppler lidar atmospheric wind sensors: a comparative performance evaluation for global measurement applications from earth orbit. Applied optics 25 (15), 2546–2553 (1986). 18231526 10.1364/ao.25.002546

[b52] Gong W., Ma X., Dong Y., Lin H. & Li J. The use of 1572 nm Mie LiDAR for observation of the optical properties of aerosols over Wuhan, China. Opt. Laser Technol. 56, 52–57 (2014).

[b53] Wojcik M. *et al.* Development of differential absorption lidar (DIAL) for detection of CO_2_, CH_4_ and PM in Alberta. Paper presented at SPIE conference 9486, Advanced Environmental, Chemical, and Biological Sensing Technologies XII, Baltimore, Maryland, United States, 20–21 April 2015. Proc. of SPIE 0277-786X, 9486, paper 9486-20, 10.1117/12.2199447 (2015 May 22).

[b54] Fiorani L., Saleh W. R., Burton M., Puiu A. & Queißer M. Spectroscopic considerations on DIAL measurement of carbon dioxide in volcanic emissions. J. Optoelectron. Adv. M. 15, 317–325 (2013).

[b55] Fiorani L. *et al.* Lidar sounding of volcanic plumes. Paper presented at SPIE conference 8894, Lidar Technologies, Techniques, and Measurements for Atmospheric Remote Sensing IX, Dresden, Germany, 23 September 2013. Proc. of SPIE 8894, paper 889407-1, 10.1117/12.2028364, (2013 October 22).

[b56] Matsushima N. & Shinohara H. Visible and invisible volcanic plumes. Geophys. Res. Lett. 33, L24309, 10.1029/2006GL026506 (2006).

