# Supplementary material for: New ground-based lidar enables volcanic CO2 flux measurements
Source: Sci Rep. 2015 Sep 1;5:13614. doi: 10.1038/srep13614 (PMC4642548; doi:10.1038/srep13614)
Supplement: Supplementary Information [file srep13614-s1.pdf]

New ground-based lidar enables volcanic

CO_2 flux measurements

Alessandro Aiuppa[1, 2]*, Luca Fiorani[3], Simone Santoro[1,4], Stefano Parracino[4,5], Marcello Nuvoli[3]

Giovanni Chiodini[6], Carmine Minopoli[7], Giancarlo Tamburello[1]

Supplementary Figures

Figure S1

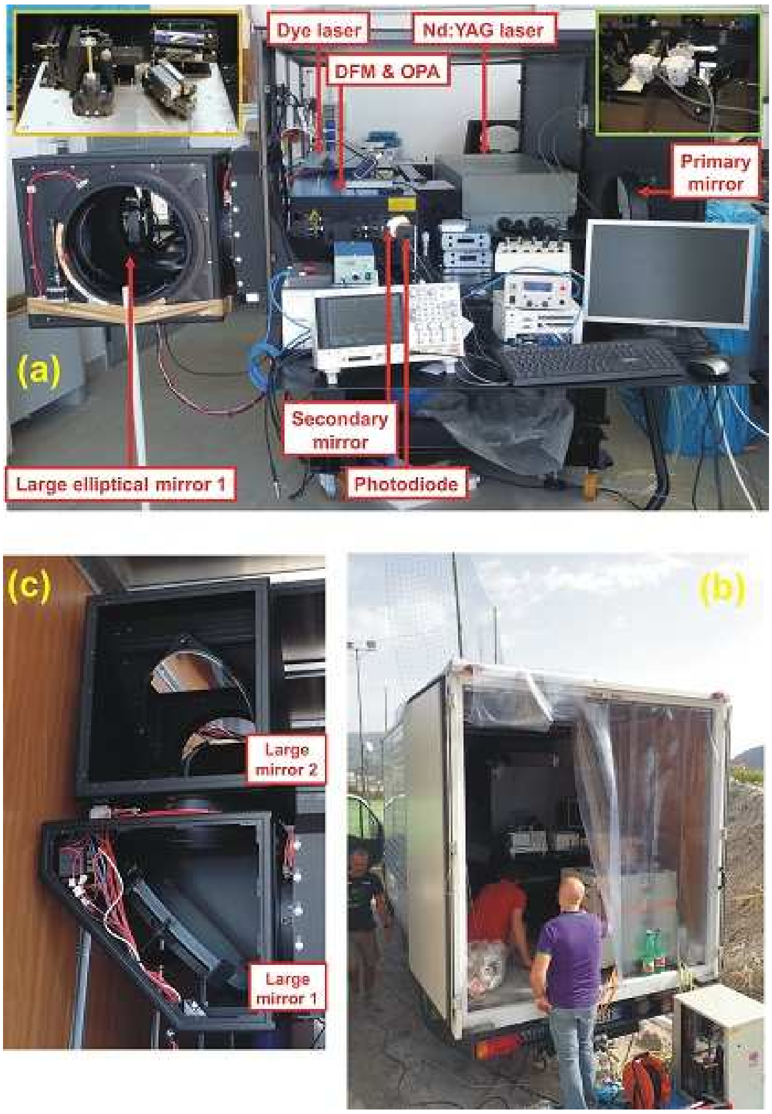

Figure S1: (a) The DIAL during preliminary tests performed at the Frascati ENEA research center. The primary and secondary mirrors of the Newtonian telescope are indicated. The orange box illustrates the operating principle of piezoelectric-element-based wavelength control: the rotatable Littrow grating is mounted on a tilting piezoelectric element (the silver block behind the grating with a diagonal slit and black cable). The green box shows one assembly of the double crystal. Only one large elliptical mirror was mounted. (b) The DIAL during the field campaign. (c) Zoomed photograph showing the large elliptical mirrors during the field campaign. All photos by C. Minopoli.

Figure S2

Figure S2: (a) Visible image of the Pisciarelli fumarolic field. (b) Brightness component of the visible image. (c) Example horizontal brightness profiles, calculated along two parallel, closely spaced (Δx=1.07 m) cross sections of the plume [*j* and *k* in (a) and (b)]. (d) Example time series of the "integrated brightness" [calculated for the temporal interval indicated by the gray shading in (e)]; temporal shift Δt between the two time series is indicated. (e) Example time series of the plume transport speed (in green), obtained by cross-correlation analysis between the "integrated brightness" time series; the cross-correlation coefficient (R^2) is indicated in blue. All photos by C. Minopoli.

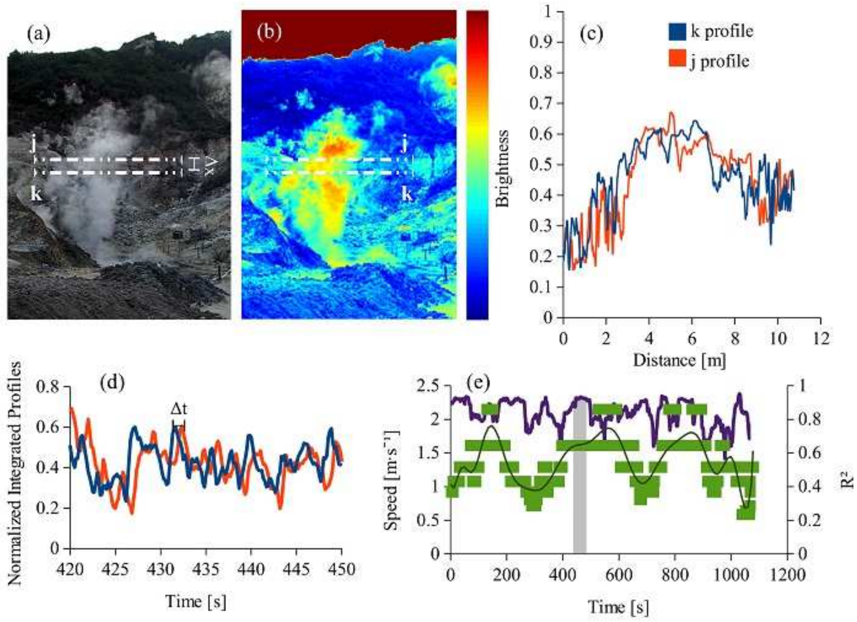

Figure S3

Figure S3: Schematic representation of the procedure used to process the lidar data. The background-corrected CO_2 mixing ratios ($C_{i\text{-corr}}$) are integrated over the entire plume cross section to obtain the total-plume CO_2 molecular density (see Methods).

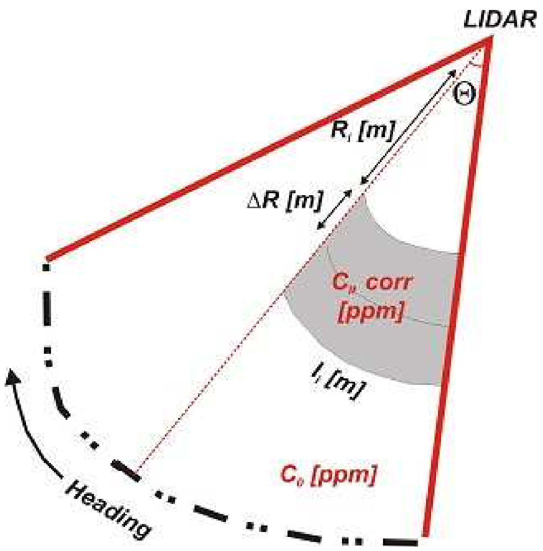

Table S1: Technical specifications of the DIAL-LIDAR used in this study

Pulse energy	25 mJ
Pulse duration	8 ns (full width at half maximum)
Repetition rate	10 Hz
Transmitted wavelength	1500 – 2100 nm
Beam divergence	0.5 mrad (full angle)
Receiver coating	Al
Receiver diameter	310 mm
Receiver focal length	0.9 m
Detector diameter	1 mm
Detector photosensitivity	1.2 A W^{-1}
Detector specific detectivity	3.5×10^{11} cm $Hz^{1/2}$ W^{-1}
Bandwidth	0 ÷ 10 MHz
Dynamic range	14 bit
Sampling rate	100 Ms s^{-1}
